# A retrospective 5-year review of rubella in South Africa prior to the introduction of a rubella-containing vaccine

**DOI:** 10.1371/journal.pone.0265870

**Published:** 2022-05-05

**Authors:** Heather Hong, Susan Malfeld, Sheilagh Smit, Lillian Makhathini, Mirriam Fortuin, Tshepo Motsamai, Dipolelo Tselana, Morubula Jack Manamela, Nkengafac Villyen Motaze, Genevie Ntshoe, Mercy Kamupira, Ester Khosa-Lesola, Sibongile Mokoena, Thulasizwe Buthelezi, Elizabeth Maseti, Melinda Suchard

**Affiliations:** 1 Centre for Vaccines and Immunology, National Institute for Communicable Diseases, National Health Laboratory Service, Johannesburg, South Africa; 2 Department of Virology, School of Pathology, Faculty of Health Sciences, University of the Witwatersrand, Johannesburg, South Africa; 3 Department of Global Health, Faculty of Medicine and Health Sciences, Stellenbosch University, Cape Town, South Africa; 4 Outbreak Response Unit, Division of Public Health Surveillance and Response, National Institute for Communicable Diseases, National Health Laboratory Service, Johannesburg, South Africa; 5 School of Health Systems and Public Health, Faculty of Health Sciences, University of Pretoria, Pretoria, South Africa; 6 World Health Organization, Pretoria, South Africa; 7 Child, Youth and School Health, National Department of Health, Pretoria, South Africa; 8 Department of Chemical Pathology, School of Pathology, University of the Witwatersrand, Johannesburg, South Africa; Health Directorate, LUXEMBOURG

## Abstract

South Africa has yet to introduce a rubella-containing vaccine (RCV) into its Expanded Programme on Immunisation (EPI). Here we evaluated the incidence of laboratory-confirmed rubella and congenital rubella syndrome (CRS) cases over the years 2015 to 2019, to document the epidemiology of rubella and CRS within South Africa prior to a RCV introduction. This retrospective study evaluated the number of laboratory-confirmed rubella cases reported through the national febrile rash surveillance system. A positive test for rubella immunoglobulin M (IgM) antibodies was considered a confirmed rubella case. For CRS cases, we reported laboratory-confirmed CRS cases collected from 28 sentinel-sites from all nine provinces of South Africa. From 2015–2019, 19 773 serum samples were tested for rubella IgM antibodies, 6 643 (33.6%) were confirmed rubella cases. Rubella was seasonal, with peaks in spring (September to November). Case numbers were similar between males (n = 3 239; 50.1%) and females (n = 3 232; 49.9%). The highest burden of cases occurred in 2017 (n = 2 526; 38%). The median age was 5 years (IQR: 3–7 years). Importantly, of females with rubella, 5.0% (161 of 3 232) of the cases were among women of reproductive age (15–44 years). A total of 62 CRS cases were reported, the mortality rate was 12.9% (n = 8), and the most common birth defect was congenital heart disease. In conclusion, rubella is endemic in South Africa. Children below the age of 10 years were the most affected, however, rubella was also reported among women of reproductive age. The baseline data represented here provides insight into the burden of rubella and CRS in South Africa prior to the introduction of a RCV, and can enable planning of RCV introduction into the South African EPI.

## Introduction

Rubella, or German measles, is generally a benign, self-limited viral infection, spread through droplet contact with the respiratory secretions of an infected person [1–3]. About two weeks after rubella exposure, a maculopapular rash occurs, first appearing on the face and usually progressing from head to foot, lasting about three days. Other signs and symptoms include a mild fever, headache, reddened eyes, post-auricular adenopathy, tiredness, cough, coryza (runny nose), and arthralgia (joint pain) [3]. Children may develop few or no symptoms, while adults may present with mild illness. Additionally, arthralgia or arthritis may occur in up to 70% of adult women with rubella. Clinically, rubella is often mistaken for measles, as such surveillance is usually conducted simultaneously for both measles and rubella.

Complications of rubella are rare and occur more often in adults than in children. However, infection during pregnancy, especially during the first trimester, can result in serious consequences such as miscarriages, stillbirth, and a constellation of severe birth defects, known as congenital rubella syndrome (CRS) [1, 3]. CRS birth defects may include hearing impairment, eye defects (cataracts, retinitis, microphthalmia, and glaucoma), heart defects (pulmonary stenosis, persistent ductus arteriosus), microcephaly, and developmental delay [4, 5]. Other late-onset CRS manifestations may include autism, diabetes mellitus, and thyroiditis.

Significantly, between 50–90% of children whose mothers are infected during their first trimester of pregnancy will suffer from CRS [4]. Worldwide, more than 100 000 infants are born annually with CRS [6]. To prevent rubella infection and reduce CRS, a safe and effective rubella vaccine is available. A single dose of the live attenuated vaccine is 95% effective and provides long-lasting immunity [7]. The vaccine is available in either monovalent or combination formulation with measles (MR), measles and mumps (MMR), or measles, mumps, and varicella (MMRV) [8, 9].

In countries that have included rubella-containing vaccines (RCV) into their national immunization programmes, there have been significant reductions in the incidence of rubella and CRS [10]. For example, in the United States, the incidence of reported cases of rubella fell sharply after the initiation of rubella immunization of young children in 1969 [11]. In 2004 rubella was declared eliminated in the United States. In 2009, rubella transmission in the World Health Organization’s (WHO) Region of the Americas was halted [12–14]. In 2012, the World Health Assembly endorsed the targeted elimination of rubella in five of six WHO regions by 2020 [15]. To date, the Region of the Americas, the United Kingdom, and Australia have eliminated rubella and CRS. However, the African Region has yet to set regional rubella targets. One of the reasons for the low uptake of RCV in the African Region has been the lack of information regarding disease burden [16]. As such, the WHO African Region has supported countries in generating information on rubella incidence via febrile rash surveillance [17].

In South Africa, a RCV is not yet part of the current South African Expanded Programme on Immunization (SA-EPI) [18]. However, within the private health sector, a RCV can be obtained as an MMR vaccine administered at 12 months and 5–6 years of age. Historically, the omission of RCV from the EPI was based on the understanding that natural rubella infection during childhood should render most women of childbearing age immune, therefore preventing CRS. In addition, under conditions of imperfect vaccine coverage, the addition of a RCV could increase the susceptibility of adult women by slowing, not interrupting, rubella transmission, thereby increasing the average age of infection, resulting in a paradoxical increase in CRS cases [19–22].

Regarding rubella in South Africa, using national surveillance data Metcalf et al. [19] reported a total of 16 466 rubella cases from 1998 to 2010 (averaging 1 266 cases per year). Thereafter, from 2013 to 2014, rubella surveillance was discontinued for South Africa and re-established in May 2015.

For CRS, the exact incidence in South Africa is not known. Through provincial sentinel site surveillance, 95 laboratory-confirmed CRS cases were reported over the period 2010 to 2017 [23]. In 2011, only 2 cases of CRS were reported the Neonatal Unit at Groote Schuur Hospital, Cape Town [24]. Over ninety percent of the mothers were young women aged between 14–30 years old, indicating an immunity gap within this age group [23]. Similarly, in a cross-sectional serosurvey for rubella immunity (detecting the presence of immunoglobulin G (IgG) antibodies), among individuals of all ages in South Africa, approximately 20% of individuals aged between 16 to 49 years old were susceptible to rubella (IgG negative) [18].

Before the introduction of a RCV into the South African EPI it is important to describe the epidemiology of rubella. Here we analysed data from the febrile rash surveillance system in South Africa over the 5 years, 2015–2019, prior to the first confirmed case of the coronavirus disease 2019 (COVID-19) on March 5^th^, 2020. We describe the epidemiology of rubella in South Africa in the public health sector before the changes in rubella epidemiology due to lockdowns and social distancing measures during 2020–2021.

## Methods

### Clinical diagnosis and case-based surveillance

As rubella symptoms appear similar to those caused by the measles virus. In South Africa, the practice is that all suspected measles cases have a blood sample taken for concurrent testing for measles and rubella IgM antibodies. The case definition for a suspected measles case per the WHO Africa region [25], is any person with generalized maculopapular rash and fever, and any one of the three C’s: cough, coryza, or conjunctivitis; or any person in whom a physician suspects measles.

For each suspected case, surveillance officers collect relevant epidemiological data, including demographic and clinical information on a case investigation form (CIF). A unique identifier or epidemiological identification (EPID) number that contains the country code, provincial code, district code, year, and the sequential case number by order of reporting (e.g. SOA-GAP-EKH-19-001) is also assigned to each suspected case. This EPID number is formulated to designate the geographic location and the year of the case under investigation and to facilitate further collection and merging of clinical, epidemiological, and laboratory data [25]. However, in some cases, a sample from a suspected case may arrive at the testing laboratory with only a lab request form without a CIF, EPID number, or both.

### Sample collection and serological testing for rubella virus IgM antibodies

Blood samples were collected from persons with febrile rash and transported to the Centre for Vaccines and Immunology, National Institute for Communicable Diseases (NICD), a division of the National Health Laboratory Services (NHLS), Johannesburg, South Africa. The laboratory is accredited by the WHO as a regional reference laboratory for measles and rubella. Sera were tested for the presence of both measles and rubella-specific IgM antibodies using commercial enzyme-linked immunosorbent assay (ELISA) kits. For the period 2015 to 2018, sera were tested using Enzygnost® Anti-Rubella Virus/IgM kit, (Siemens AG, Erlangen, Germany). For the period 2018 to 2019, sera were tested using Euroimmun Anti-Rubella Virus ELISA (IgM), (Euroimmun AG, Luebeck, Germany). A positive serologic test for rubella IgM antibody was considered a laboratory-confirmed rubella case. Data from private sector laboratories were not included.

### Surveillance indicators

Surveillance performance was assessed using the standard indicators recommended in the WHO African regional guidelines for measles and rubella surveillance [25]. The primary indicator used to assess the sensitivity and representativeness of the case-based surveillance system was the number of discarded cases per year, i.e. the non-measles, non-rubella febrile cases per 100,000 population, with a target rate of at least 2 cases of febrile rash notified per 100,000 population per year.

### Congenital rubella syndrome surveillance

In 2015, sentinel-site surveillance for CRS was established at 28 clinical sites and 6 laboratories within the 9 South African provinces and data up until 2017 has been previously reported [23]. In brief, at each site infants aged less than 12 months who had presented with at least one CRS compatible symptoms (i.e. cataract, congenital glaucoma, congenital heart disease, hearing impairment, pigmentary retinopathy, purpura, hepatosplenomegaly, jaundice, microcephaly, developmental delay, meningoencephalitis, or radiolucent bone disease), and who had tested positive by rubella IgM or two serial rubella IgG tests at least 4 weeks apart with titres that did not drop 2-fold or positive rubella PCR at various testing laboratories were included [23]. At the end of each month, focal clinicians and the NHLS virology departments were contacted and requested to share information on any laboratory-confirmed CRS cases and complete a CRS CIF [23].

### Ethics

For rubella, febrile rash surveillance was part of the routine surveillance by the NICD with Human Research Ethics Committee (Medical), University of the Witwatersrand (M160667). For CRS, ethical approval was obtained from all 9 provincial ethics committees as well as university research ethics covering the tertiary hospitals [23].

### Data analysis

Rubella and CRS case numbers were based on their date of onset, date of sample collection if the date of onset was not available, or date of notification. For both rubella and CRS, descriptive analyses were performed using Microsoft Excel 2016. Continuous variables were reported using medians and ranges while categorical variables were reported using absolute numbers and percentages. The annual incidence was calculated by dividing the number of cases in a given year by the mid-year population estimates for South Africa [26].

## Results

Due to rubella testing restrictions in 2015 (1 January to 5 May), 1 263 suspected cases with febrile rash were not tested for rubella IgM. Thereafter, from 6 May 2015 to 31 December 2019, 20 056 febrile rash cases were received from all 9 provinces in South Africa. A total of 19 773 (98.6%) samples were tested, 283 (1.4%) were rejected due to insufficient sample volume or inappropriate sample type, 11 716 (59.3%) had a negative test result, 1 414 (7.2%) had an equivocal test result and 6 643 cases (33.6%) were positive and were confirmed as rubella cases (**Table 1**).

**Table 1:** Distribution of suspected and confirmed rubella cases, 2015–2019.

Year	Febrile rash cases	Total tested for rubella	Number (%) positive for rubella	Number (%) equivocal for rubella	Number (%) negative for rubella
**2015**	2 595	2 594	572 (22.1%)	137 (5.3%)	1 885 (72.7%)
**2016**	2 820	2 810	821 (29.2%)	182 (6.5%)	1 807 (64.3%)
**2017**	6 272	6 159	2 526 (41.0%)	450 (7.3%)	3 183 (51.7%)
**2018**	3 761	3 710	1 228 (33.1%)	284 (7.7%)	2 198 (59.2%)
**2019**	4 608	4 500	1 496 (33.2%)	361 (8.0%)	2 643 (58.7%)
**Total**	20 056	19 773	6 643 (33.6%)	1 414 (7.2%)	11 716 (59.3%)

**Fig 1:**
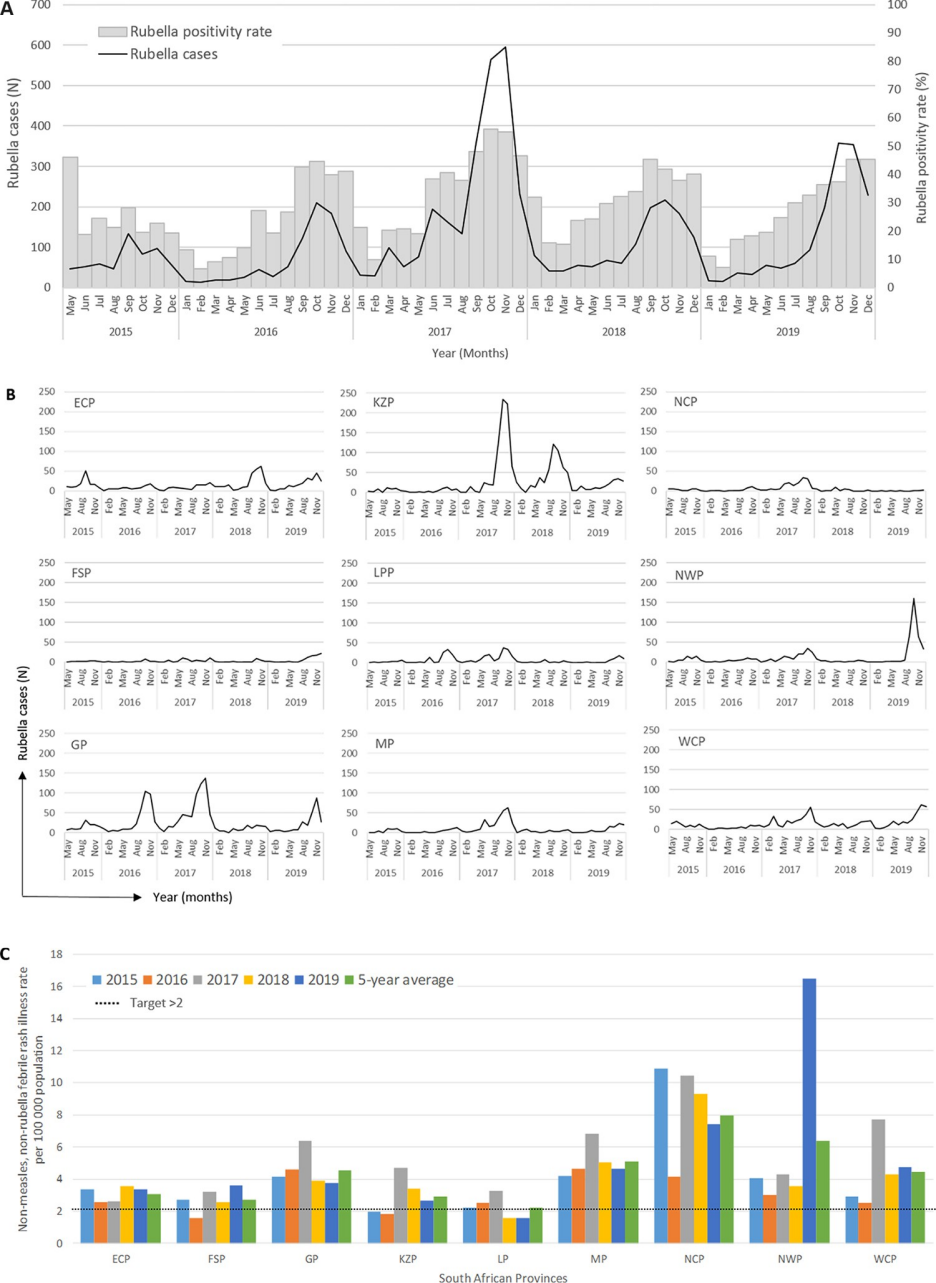
Rubella in South Africa from 2015–2019. **A)** Monthly distribution of confirmed rubella cases and rubella positivity rate amongst febrile rash samples, **B)** Monthly distribution of confirmed rubella cases within each of the South African provinces, **C)** Provincial non-measles, non-rubella febrile rash illness rate per 100 000 population, the target was at least 2 cases per 100 000 population should be met. **ECP**, Eastern Cape Province; **FSP**, Free State Province; **GP**, Gauteng Province; **KZP**, KwaZulu-Natal province; **LPP**, Limpopo province; **MP**, Mpumalanga Province; **NCP**, Northern Cape Province; **NWP**, northwest province; **WCP**, Western Cape Province.

Rubella circulated widely in South Africa, peaking in the spring months from September to November (**Fig 1A**). Over the five years, the annual average was 1 329 rubella cases per year. The highest number of rubella cases occurred in 2017 (2 526; 38%) followed by 2019 (1 496; 22.5%). In terms of the proportion of cases in South Africa **(Fig 1B)**, KwaZulu-Natal Province had the highest burden of cases (1 559, 23.5%) followed by Gauteng Province (1 492, 22.5%). Of note, in KwaZulu-Natal Province, there was a 14-fold increase in rubella case numbers from 53 cases in 2016 to 730 cases in 2017. The Free-State province had the least number of rubella cases (163, 2.5%).

Over the five years, the average non-measles, non‑rubella febrile rash illness rate was 3.98 per 100 000, and the confirmed rubella incidence rate was 23.4 per million population (**S7 Table**). The highest rates were in 2017, amounting to 5.3 non-measles, non‑rubella febrile rash illnesses per 100 000 and 44.4 confirmed rubella cases per million population. When reviewing provincial data, in 2017 the Northern Cape province had the highest confirmed rubella incidence rate of 139.2 per million population.

For the non-measles, non‑rubella febrile rash illness rate, a target of at least 2 per 100 000 population, is indicative of adequate surveillance. When reviewing the five-year provincial average, this target was met by all nine provinces. Overall, the national average reached 4 per 100 000 population, with the Northern Cape province performing the best achieving 8 per 100 000 population. At the provincial level, however, some provinces failed to meet the target of at least 2 non-measles, non‑rubella febrile rash cases (Free State and Kwa-Zulu Natal provinces in 2016, and the Limpopo province in 2018 and 2019, **Fig 1C**).

**Fig 2:**
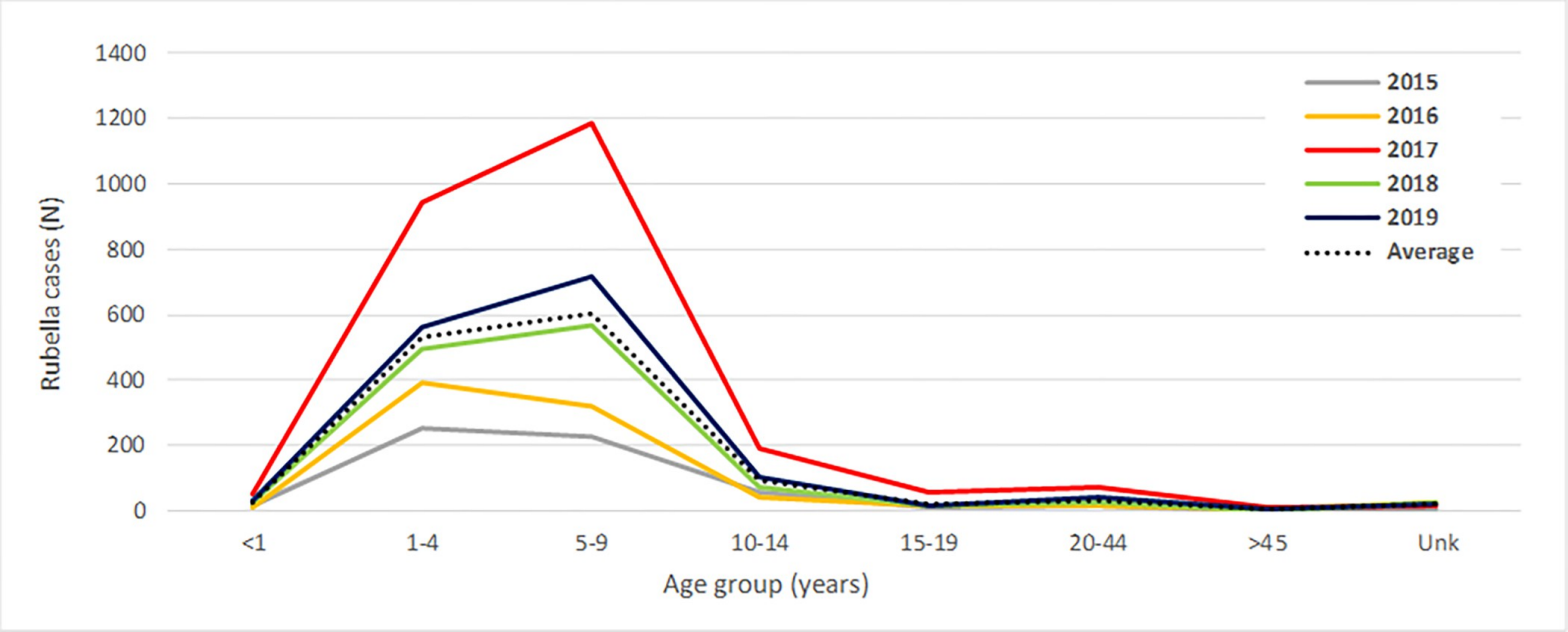
Age distribution of rubella cases, 2015–2019.

Of the confirmed rubella cases, 96.2% (6 393 of 6 643) had information on age and gender. The median age for rubella infection was 5 years (IQR: 3–7 years). There were more 5–9 year olds (45.4%) than 1–4 year olds (39.8%) amongst the cases (**Fig 2**). Rubella was similarly distributed amongst males (3 239, 50.1%) and females (3 232; 49.9%). Notably of the females with rubella, 5.0% (161 of 3 237) of the cases were among women of reproductive age (15–44 years).

Regarding surveillance indicators (**S2 Table**), of the total laboratory-confirmed rubella cases (n = 6 643), 3 769 (57.8%) had a CIF, 3 483 (52.4%) had a unique EPID number, and 2 691 (40.7%) had both a CIF and EPID number. The worst performance in terms of CIF accompaniment (33%), EPID number allocation (22.4%), and the combination CIF and EPID number (13.8%) was in 2016. Thereafter, there was some improvement from 2017 onwards but rates remained below 50%, highlighting areas of surveillance that require significant improvement.

Through sentinel site surveillance [23], a total of 62 CRS cases were reported, originating from all provinces except North West Province (**Table 2**). CRS cases were the highest in 2015 (n = 37) and steadily declined thereafter, with the lowest number of CRS reported in 2019 (n = 4). At the provincial level, Western Cape province had the highest number of CRS cases (33.9%), followed by Gauteng (19.4%) and Free State province (14.5%).

**Table 2:** Sentinel site surveillance for congenital rubella syndrome (CRS), South Africa, 2015–2019.

Province	2015*	2016*	2017*	2018	2019	Provincial total
**ECP**	4	0	0	1	0	5
**FSP**	6	0	2	1	0	9
**GP**	0	7	4	1	0	12
**KZP**	3	0	0	0	0	3
**LP**	3	0	2	0	0	5
**MP**	2	0	0	0	0	2
**NCP**	1	0	0	0	0	1
**NWP**	0	0	0	0	0	0
**WCP**	18	1	0	2	0	21
**Unknown**	0	0	0	0	4	4
**Total**	37	8	8	5	4	62

* Previously published CRS case numbers by Motaze et al. [23].

Combining our data with that published by Motaze et al. [23], of 62 infants with CRS 33 (53%) were female, 29 (47%) were diagnosed within the first month of birth, and the most common birth defect was congenital heart disease (77.4%), primarily presenting as patent ductus arteriosus, followed by cataracts (48.4%) and hepatosplenomegaly (43.5%), **Table 3**. At the time data was captured, 12.9% of the reported CRS cases had died.

**Table 3:** Infant and maternal characteristics of confirmed congenital rubella syndrome cases identified at sentinel surveillance sites, South Africa, 2015–2019, N = 62.

**Infant Characteristics**	**N**	**%**
Sex		
Females	33	53.2
Males	27	43.5
Unknown	4	6.5
Age at diagnosis		
< 1 month	29	46.8
1–5 months	27	43.5
> 5 months	2	3.2
Unknown	4	6.5
Birth defects		
Cataracts	30	48.4
Congenital heart disease	47	75.8
Glaucoma	2	3.2
Hearing impairment	3	4.8
Hepatosplenomegaly	27	43.5
Jaundice	12	19.4
Meningoencephalitis	8	12.9
Mental retardation	2	3.2
Microcephaly	27	43.5
Pigmentary retinopathy	2	3.2
Purpura	15	24.2
Radiolucent bone disease	6	9.7
Mortality		
Alive	43	69.4
Died	8	12.9
Unknown	11	17.7
**Maternal Characteristics**	**N**	**%**
Age (median (Range)	22 years (15–38)	
Age group		
10–14	0	0
15–19	11	17.7
20–45	34	54.8
> 45	0	0
Not available	17	27.4
Parity		
1	22	35.5
≥ 2	21	33.9
Unknown	19	30.6
Clinical manifestations		
Arthralgia/ arthritis	3	4.8
Conjunctivitis	2	3.2
Lymph node swelling	0	0
Rash during pregnancy	8	12.9
None	13	21.0
Unknown	38	61.3

Unknown refers to CRS cases that had clinical information unavailable for certain variables.

Regarding maternal characteristics, the median age was 22 years old (range: 15–38 years). The median parity was one (range: 1–4), and the median gestation was 36.5 weeks (range: 31–41 weeks). Clinical manifestations of rubella infection in mothers were uncommon, only 13% reported the presence of a maculopapular rash during pregnancy. As rubella vaccination is not yet part of the EPI, the majority of mothers did not know their rubella vaccination status (79.1%).

## Discussion and conclusion

Using the national febrile rash surveillance data from 2015 to 2019, we have shown that rubella is endemic and circulates widely in South Africa. On average there were 1 329 rubella cases per year with seasonal peaks in spring (September-October). The average incidence rate was 23.4 per million population, varying considerably between provinces. Limpopo province had the lowest incidence rate of 3.3 cases per million in 2015 and Northern Cape province had the highest incidence rate of 139.2 cases per million in 2017.

The national epidemic curve (**Fig 1A**) suggested rubella outbreaks in 2017 and 2019. Notably at the provincial level, outbreaks occurred within specific provinces and at different intervals (**Fig 1B**). In agreement, rubella outbreaks were declared at two schools in 2017; one in Tshwane, Gauteng province, and the other in the Overberg, Western Cape province [27]. In the Tshwane district, 36 rubella cases were identified (29 were learners and 7 were facilitators). In the Overberg district, 75 school learners were affected. Rubella outbreaks often happen in settings such as schools and hospitals, in which nonimmune individuals are in close contact with a case [28]. The occurrence of these outbreaks in South Africa, in the absence of a public RCV, may be associated with the build-up of susceptible persons in the population as well as high contact rates [3]. While the 5-year surveillance period is short, the national epidemic curve suggests that rubella outbreaks follow a two-year cycle, which is more frequent than the pre-vaccine outbreaks reported in the United States (every 6 to 9 years) or in Europe (every 3 to 5 years) [29], suggesting that rubella susceptibility is high in South Africa. Our five-year interval may be too short to clearly depict the frequency of outbreaks at provincial or district level.

Young children under the age of 10 years were the most affected (87%). For the years 2015 and 2016, the 1–4 year olds had the higher case numbers, compared to the years 2017 to 2019 in which 5–9 year olds had higher case numbers. This subtle shift towards the 5-9 year olds should be closely monitored as a shift to older age groups may impact RCV introduction strategies and/or catch-up campaigns. Notably, over the 5 years, there were 161 cases of rubella in women of childbearing age, the highest percentage occurring in 2017 (45%). Unfortunately, there was no further follow-up on these women, and it remains unknown if they may have been pregnant at the time of diagnosis, or if they were informed or advised about the possible consequences of rubella infection during pregnancy. WHO recommends that once rubella infection is identified there be prompt case follow-up [30]. The assigned case investigation officer should determine if the patient is pregnant, whether they have close contacts that are female of childbearing age or are pregnant, counsel the patient regarding the risks of CRI and CRS, if the patient is pregnant, the patient should be registered on a CRI/CRS pregnancy registry to allow for tailored medical follow-up, and infants born to mothers with rubella should be tested for rubella-specific IgM [30].

Over the 5 years, which included data previously reported by Moatze et al. [23] and our data from 2018–2019, 62 confirmed cases of CRS were reported. The annual average was 12 CRS cases per year. Reported CRS case numbers decreased from 37 cases in 2015 to 4 cases in 2019. Mortality amongst the CRS cases was above 10%. A large proportion of infants presented with congenital heart disease (77%) or cataracts (48%). Other conditions, such as hearing impairment and mental retardation were detected in less than 5% and 3% of the CRS cases, respectively. While these clinical frequencies are similar to other reports [16], the CRS case numbers are likely grossly underestimated. Schoub et al. [22] predicted that there should be approximately 654 cases of CRS per year in South Africa. There are could be several reasons for the vast difference in case numbers reported in 2015 through 2019. Firstly, the higher caseload may be attributed to heightened awareness by clinicians for CRS during the first year. Secondly, many cases may not be detected and/or reported by the surveillance system perhaps due to the asymptomatic nature of rubella infection in mothers, the rarity and subtle nature of CRS symptoms in infants and children, some clinical manifestations may not be present at birth or only present later in life, (e.g. deafness or mental retardation), only severe cases may be diagnosed [23, 31]. Additionally, as CRS sentinel site surveillance is not yet part of the national rash surveillance system, it was not designed to capture all cases, but rather to monitor trends and form a recent baseline for CRS in South Africa.

To address these issues and to improve CRS surveillance, additional sentinel-sites may be added to increase case finding; more frequent communication with the CRS focal groups could be set up; annual CRS meetings could be held between the sentinel sites and the CRS surveillance team, wherein retraining on case finding, the CRS case reporting procedure, and rubella and CRS case numbers can be discussed; in addition, active facility-based surveillance for CRS should be conducted locally following rubella outbreaks to detect CRS in the infant cohorts.

To measure the adequacy of rubella case-finding activity taking place in South Africa, the WHO surveillance target of at least 2 non-measles, non-rubella febrile rash illness per 100 000 population was used. Overall, South Africa successfully met this target each year, with a five-year average of 4 non-measles, non-rubella febrile rash cases per 100 000 population. This surveillance indicator effectively doubled over the 5 years, suggesting good performance in the national rash-based surveillance system. However, of concern, was the lack of CIFs (57.8%), EPID number assignment (52.4%) as well as poor completion or illegible CIFs. The CIF and EPID are critical to the epidemiological monitoring, ensuring timely actions are met as well as identifying if there were complications or hospitalization. Partial and/or missing CIFS and EPID numbers hinder case investigation and will impede evaluation of vaccine efficacy once a RCV is introduced. These areas need significant improvement. One option would be to utilize an electronic data capturing system to increase efficiency, and enhance data quality. An electronic notifiable medical conditions system recently established in South Africa may alleviate some difficulties although currently only allows for electronic case reporting rather than detailed case investigation.

Our findings provide baseline data for a rubella control programme in South Africa. Given that humans are the only known host for the rubella virus, that rubella is less contagious than measles, that available rubella vaccines are safe and highly effective, that the national febrile rash-based surveillance system is meeting WHO performance indicators and the testing laboratories have accurate diagnostic assays to detect rubella infection, it is possible to set a goal for rubella and CRS elimination in South Africa. Such a strategy would likely involve the implementation of combined vaccination of male and female infants, as well as boosting of rubella immunity before sexual maturity and/or a catch-up campaign for older children, to avoid paradoxically increasing rubella and CRS incidence [3, 13]. Simply vaccinating young women only would not eradicate CRS unless 100% were immunised and is not recommended by the WHO. Recently various scenarios have been modelled [32, 33]. Comparing different vaccine strategies and their relative costs, scenarios including mass campaigns resulted in more rapid elimination of rubella and CRS, however routine vaccination at 12 months of age coupled with vaccination of nine-year-old children was associated with the lowest cost per CRS case averted for a similar percentage reduction in CRS [33]. At 80% RCV coverage, all vaccine introduction scenarios modelled, would achieve rubella and CRS elimination in South Africa [32].

In South Africa, the National Department of Health reported that the measles 1^st^ dose national immunisation coverage was 77.0% and the 2nd dose coverage was 76.4% [34]. This is encouraging and suggests that South Africa may be ready for RCV implementation, in the form of an MR vaccine, however confirmation of coverage figures await results from the ongoing national vaccine coverage survey [35]. Failure to introduce an RCV into the national EPI will see rubella outbreaks frequently occur; however, improper introduction RCV may result in pushing the epidemic into older age groups, both scenarios resulting in probable CRS cases.

In conclusion, using the national febrile rash and CRS sentinel-site surveillance data we show that rubella and CRS are significant health concerns in South Africa. The data present here can be used for epidemiological modelling and to inform RCV immunisation options, such as the inclusion of various age groups, targeting young girls and/or boys, as well as catch-up campaign strategies. Furthermore, to monitor the impact of a RCV, this study highlights the need for improvements to both surveillance systems. For the national febrile rash-based system, each case should also have a completed CIF and an EPID number, moreover, rubella outbreaks should be investigated and documented. For CRS sentinel-site surveillance, the decrease in reported CRS case numbers underscores the urgent need for heightened newborn screening. Future improvements may also include the use of routine characterization of rubella genotypes. Molecular epidemiology allows the differentiation of circulating rubella viruses, can be used to monitor the transmission pathways during outbreak investigations and to identify interruption of endemic virus transmission. Thus, we provide rubella and CRS data and methods for improving surveillance in preparation for the RCV into the SA-EPI.

## Supporting information

S1 Table Rubella incidence rate per million population by provinces in South Africa, 2015–2019. For non-measles, febrile rash illness rate per 100 000, green indicates good performance by meeting the WHO surveillance target and red indicates poor performance i.e. not meeting the surveillance target. ECP, Eastern Cape Province; FSP, Free State Province; GAP, Gauteng Province; KZP, KwaZulu-Natal province; LPP, Limpopo province; MPP, Mpumalanga Province; NCP, Northern Cape Province; NWP, northwest province; WCP, Western Cape Province. *missing one sample, not designated to a province.
(XLSX)

S2 Table Surveillance indicators for laboratory-confirmed rubella cases in South Africa, 2015-2019. CIF: case investigation form; EPID: unique epidemiological number.
(XLSX)

S3 Table National febrile rash surveillance data in South Africa for 2015 (N = 2595). ECP, Eastern Cape Province; FSP, Free State Province; GAP, Gauteng Province; KZP, KwaZulu-Natal province; LPP, Limpopo province; MPP, Mpumalanga Province; NCP, Northern Cape Province; NWP, northwest province; WCP, Western Cape Province.
(XLSX)

S4 Table National febrile rash surveillance data in South Africa for 2016 (N = 2820). ECP, Eastern Cape Province; FSP, Free State Province; GAP, Gauteng Province; KZP, KwaZulu-Natal province; LPP, Limpopo province; MPP, Mpumalanga Province; NCP, Northern Cape Province; NWP, northwest province; WCP, Western Cape Province.
(XLSX)

S5 Table National febrile rash surveillance data in South Africa for 2017 (N = 6272). ECP, Eastern Cape Province; FSP, Free State Province; GAP, Gauteng Province; KZP, KwaZulu-Natal province; LPP, Limpopo province; MPP, Mpumalanga Province; NCP, Northern Cape Province; NWP, northwest province; WCP, Western Cape Province.
(XLSX)

S6 Table National febrile rash surveillance data in South Africa for 2018 (N = 3761). ECP, Eastern Cape Province; FSP, Free State Province; GAP, Gauteng Province; KZP, KwaZulu-Natal province; LPP, Limpopo province; MPP, Mpumalanga Province; NCP, Northern Cape Province; NWP, northwest province; WCP, Western Cape Province.
(XLSX)

S7 Table National febrile rash surveillance data in South Africa for 2019 (N = 4608). ECP, Eastern Cape Province; FSP, Free State Province; GAP, Gauteng Province; KZP, KwaZulu-Natal province; LPP, Limpopo province; MPP, Mpumalanga Province; NCP, Northern Cape Province; NWP, northwest province; WCP, Western Cape Province.
(XLSX)

S8 Table Infant and maternal characteristics of reported laboratory-confirmed congenital rubella syndrome cases identified at sentinel surveillance sites, South Africa, 2015–2019 (N = 62). (XLSX)
